# The Epidemiology of Ascites in a Multi‐Ethnic Asian Population

**DOI:** 10.1002/jgh3.70111

**Published:** 2025-02-13

**Authors:** Ram Prasad Sinnanaidu, Kumaraganapathy Poobalan, Aswinderjeet Singh Balwan Singh, Kishvan Nair, Anushya Vijayananthan, Sanjiv Mahadeva

**Affiliations:** ^1^ Division of Gastroenterology, Department of Medicine, Faculty of Medicine University of Malaya Kuala Lumpur Malaysia; ^2^ Department of Biomedical Imaging, Faculty of Medicine University of Malaya Kuala Lumpur Malaysia

**Keywords:** ascites, Asians, cirrhosis, congestive cardiac failure, malignancy

## Abstract

**Introduction:**

Ascites is a common condition seen by clinicians in secondary care. Data on the epidemiology of ascites in Asians is lacking.

**Methodology:**

A retrospective case record review was performed in this large, referral institution between January 2016 and December 2019. Clinical and epidemiological data of adult (age > 18 years) patients with ascites, identified from the Radiology database, were obtained from this institutions' electronic medical records.

**Results:**

A total of 838 patients (median age 59.77 ± 14.46 years, 56% males, ethnicity: Chinese 41.9%, Malay 34.8%, Indian 22.7%) were included in the study. Malignancy (28.9%) and liver cirrhosis (27.9%) were the most common etiology of ascites. Most of the malignant etiology of ascites were due to female‐related (breast and ovarian) and gastrointestinal (colon, liver, pancreatic, bile duct) cancer. Liver cirrhosis‐related ascites was mostly due to metabolic‐associated fatty liver disease (MASLD, 35.5%) and hepatitis B infection (20.5%). An increased age (> 40 years) was associated with all causes of ascites. The etiology of ascites varied with ethnicity as follows: the most common cause of ascites was malignancy (37.6%) among ethnic Chinese, heart failure (20.5%) in ethnic Malays and chronic liver disease (43.7%) in ethnic Indians.

**Conclusion:**

Malignancy and liver cirrhosis are the leading cause of ascites in a multi‐ethnic Asian population. Demographic factors, particularly ethnicity, have a strong influence on the etiology of ascites.

## 1 Introduction

Ascites is defined as a pathological accumulation of fluid within the peritoneal cavity. Physiologically, there is no intraperitoneal fluid observed in males, while females tend to have up to 20 mL of intraperitoneal fluid due to their menstrual cycle [1].

There are several pathophysiological mechanisms for the development of ascites. The commonest pathophysiology is the development of portal hypertension. The increment of portal venous pressure, aka portal hypertension, leads to vasodilation due to an increased production of circulating nitric oxide. As vasodilation worsens, the plasma levels of vasoconstrictor sodium‐retentive hormones increase, leading to ascites formation. Another mechanism is due to the production of proteinous fluid by tumor cells lining the peritoneum in malignancy. When effective arterial blood volume is compromised for any reason, vasopressin, renin‐aldosterone and the sympathetic nervous system will be activated. This will lead to renal vasoconstriction and sodium and water retention, with the resultant formation of ascites [1]. Another mechanism involves reduced hepatic protein synthesis, which leads to hypoalbuminemia and lowering intravascular oncotic pressure, which contributes to the formation of ascites [2]. In conditions such as tuberculosis, ascites develops secondary to “exudation” of proteinaceous fluid from the tubercles, similar to the mechanism leading to ascites in patients with peritoneal carcinomatosis [3]. Ascites has been associated with a significant reduction in a patients' quality of life and a direct implication on their disease outcome. Mortality resulting from ascites and its complications, such as spontaneous bacterial peritonitis and hepatorenal syndrome, ranges from 15% a year to 44% in 5 years [1]. Involvement of multiple systems contributing to the occurrence of ascites is not uncommon and this significantly increases the challenge of proposing a treatment plan for a patient.

Data on the epidemiology of ascites has been derived from reports in Western/Caucasian patients predominantly. The most common cause of ascites is reported to be liver cirrhosis followed by malignancy, heart failure, renal failure, tuberculosis, and nephrotic syndrome [4, 5]. The prevalence of cirrhosis, malignancy, heart failure, tuberculosis, and nephrotic syndrome is approximately 81 000, 10 000, 3000, 2000, and 1000 per 100 000 individuals with ascites, respectively [6]. However, geographical variation in the prevalence of malignancy [7], chronic liver disease [8] and tuberculosis [9] may result in a variation of the epidemiology of ascites in non‐Western populations. Furthermore, epidemiological changes in the etiology of chronic liver disease (declining viral Hepatitis and increasing metabolic dysfunction‐associated steatotic liver disease) [10], and malignancy [11], together with the declining incidence of TB (due to improved public health measures and access to treatment) [12] may influence the etiology of ascites.

The population of Malaysia is a melting‐pot of major ethnic groups from Asia, namely ethnic Chinese, ethnic Indians and those indigenous to the South East Asian region [13]. This study was conducted to obtain clinical and demographic data on ascites from a representative urban population in Malaysia, with the aim of providing further data on the epidemiology of ascites among Asian adults.

## 2 Methodology

### 2.1 Study Design

We conducted a retrospective case record review of adults (age > 18 years) with a diagnosis of ascites presenting to this large, referral institution between January 2016 to December 2019. Patients with confirmed ascites, based on ultrasound imaging of the abdomen, were identified from this institution's Radiology database. Clinical and demographical data of patients with ascites were subsequently obtained from the institutions' electronic medical records.

### 2.2 Statistical Analysis

Data were collected and analyzed using Statistical Packages for the Social Sciences (SPSS) version 26.0. The primary objective was to describe the etiology and demography of adults with ascites. A secondary objective was to explore the role of age, gender and ethnicity on the etiology of ascites. Continuous data were expressed as means (±SD) or median (with range) where appropriate, while categorical data were presented as frequency or proportions. Simple descriptive statistics were performed for both continuous data (independent *T*‐test) and categorical (chi‐square test).

A significance level of *p* ≤ 0.05 was used for all models.

### 2.3 Results

Data for a total of 838 patients with a diagnosis of ascites between the January 1, 2016 until December 31, 2019 were retrieved. Patients included into this study are within the range of 18–90 years old with the median age of 59.77 ± 14.46. There were significantly more male subjects (*n* = 469, 56%). The majority of the patients were ethnic Chinese (*n* = 351, 41.9%), followed by ethnic Malay (*n* = 292, 34.8%) and ethnic Indian (*n* = 190, 22.7%).

Overall, malignancy was the commonest cause of ascites (*n* = 242, 28.9%), followed by liver cirrhosis (*n* = 234, 27.9%), congestive cardiac failure (*n* = 141, 16.8%), and renal failure (*n* = 128, 15.3%). Less common causes of ascites were present in *n* = 93 (11.1%)—see Table 1.

**TABLE 1:** Basic demography and etiology of ascites.

Median age ± SD	59.77 ± 14.46
Male: female ratio	469:369
Ethnic distribution	*n* (%)
Malay	292 (34.8)
Chinese	351 (41.9)
Indian	190 (22.7)
Others	5(0.6)
Etiology	*n* (%)
Malignancy	242 (28.9)
Liver cirrhosis	234 (27.9)
Congestive cardiac failure	141 (16.8)
Renal failure	128 (15.3)
Pancreatitis	42 (5.0)
Tuberculosis	13 (1.6)
Dengue fever	13 (1.6)
Acute liver failure	13 (1.6)
Non‐cirrhotic portal hypertension	10 (1.2)
Hypoalbuminemia due to chronic sepsis	1 (0.1)
Hypoalbuminemia due to protein losing enteropathy	1 (0.1)

### 2.4 Sub‐Classification of Ascites Etiology

Further details of the two most common aetiologies of ascites, namely malignancy and liver cirrhosis, were explored. Among the 37 types of malignancy identified in our patients, the six most common types were breast carcinoma, *n* = 45 (18.6%); colon carcinoma, *n* = 37 (15.29%); hepatocellular carcinoma, *n* = 32 (13.22%); pancreatic carcinoma, *n* = 18 (7.44%); cholangiocarcinoma, *n* = 15 (6.2%); and ovarian carcinoma, *n* = 13 (5.37%).

For liver cirrhosis, the most common etiology identified among our patients with ascites were non‐alcoholic fatty liver disease (*n* = 83, 35.5%), followed by hepatitis B infection (*n* = 48, 20.5%), alcoholic liver disease (*n* = 45, 19.2%), hepatitis C infection (*n* = 27, 11.5%), and cryptogenic (*n* = 22, 9.4%). Less common causes of cirrhosis included autoimmune diseases (*n* = 7, 3%), biliary atresia (*n* = 1, 0.4%), and sarcoidosis (*n* = 1, 0.4%).

### 2.5 Demographic Associations With Ascites Aetiologies

In a subgroup analysis, we further examined the role of age, gender and ethnicity on the etiology of ascites. Among our patients with ascites, most of the etiologies were more common in adults aged > 40 years (Table 2). However, there was a greater proportion of malignancy among patients of female gender (*n* = 122, 50.4%) and of Chinese ethnicity (*n* = 132, 54.5%). Liver cirrhosis was more prevalent among male adults (*n* = 147, 62.8%) and those of Chinese (*n* = 83, 35.5%) and Indian (*n* = 83, 35.5%) ethnicity. Congestive cardiac failure was more common in males (*n* = 95, 67.4%) and those of Malay ethnicity (*n* = 81, 33.5%). Renal failure was more prevalent in females (*n* = 71, 55.5%) and those of Chinese ethnicity (*n* = 58, 45.3%). Details of these are summarized in Table 2.

**TABLE 2:** Etiology of ascites based on age, gender, and ethnicity.

	Age			Gender			Ethnicity				
	≤ 40 *n* = 86	> 40 *n* = 752	*p*	Male *n* = 469	Female *n* = 369	*p*	Malay *n* = 292	Chinese *n* = 351	Indian *n* = 190	Others *n* = 5	*p*
Malignancy	19 (22.1%)	223 (29.7%)	0.143	120 (25.6%)	122 (33.1%)	0.018	81 (27.7%)	132 (37.6%)	28 (14.7%)	1 (20%)	< 0.001
Liver cirrhosis	11 (12.8%)	223 (29.7%)	0.001	147 (31.3%)	87 (23.6%)	0.013	67 (22.9%)	83 (23.6%)	83 (43.7%)	1 (20.0%)	< 0.001
Congestive cardiac failure (CCF)	8 (9.3%)	133 (17.7%)	0.049	95 (20.3%)	46 (12.5%)	0.03	60 (20.5%)	49 (14.0%)	32 (16.8%)	0	0.113
Renal failure	17 (19.8%)	111 (14.8%)	0.221	57 (12.2%)	71 (19.2%)	0.05	50 (17.1%)	58 (16.5%)	19 (10.0%)	1 (20.0%)	0.147
Others	31 (36.0%)	62 (8.2%)	< 0.001	50 (10.7%)	43 (11.7%)	0.650	34 (11.6%)	29 (83%)	28 (14.7%)	2 (40.0%)	0.021

*Note:* These include pancreatitis, tuberculosis, dengue fever, acute liver failure, non‐cirrhotic portal hypertension, hypoalbuminemia due to chronic sepsis, and protein losing enteropathy.

## 3 Discussion

In this descriptive report, malignancy and liver cirrhosis were identified as the two leading etiologies of ascites in a multi‐ethnic Asian population. Our findings are somewhat different from most published reports, which have identified liver cirrhosis as the most common cause of ascites. In a study conducted in Qatar, liver cirrhosis was reported to be the most prevalent cause of ascites accounting for 62% of its cases followed by malignant ascites (12%), malignancy‐related ascites (10%), tuberculosis (8%), heart failure (7%), nephrotic syndrome (3%), chylous ascites (1%), and eosinophilic ascites (1%) [14]. Similarly, studies from the United States [1, 5] and Ethiopia [15] have shown a predominance of chronic liver disease as the main cause of ascites. Meanwhile in a study from China, both liver cirrhosis (30.3%) and malignant neoplasms (24.2%) were identified as the two most common causes of ascites [5]. The data from the Chinese study is not too different to our data, which is largely due to the disease burden of malignancy in our Malaysian population, which is significantly greater than chronic liver disease [16].

The types of malignancy that led to the development of ascites in our study was predominantly female related (breast and gynecological) and gastrointestinal (colon, liver, pancreas, and bile duct) malignancy. Previous reports of common malignancy‐related etiologies of ascites have primarily show the following—ovarian cancer followed by colorectal, pancreatic, and uterine; extra‐abdominal tumors originating from lymphoma, lung, and breast [17, 18]. It appears that the types of malignancy‐related ascites observed in our Malaysian population are similar to these published reports. With regards to cirrhosis, the commonest etiology of cirrhosis were metabolic dysfunction‐associated steatotic liver disease (MASLD), followed by hepatitis B, chronic alcohol consumption, and hepatitis C [19, 20]. These observations concur with liver disease etiology patterns from other published studies on ascites [14, 15].

The exploration of demographic factors highlighted that an increased age was significantly associated with most etiologies of ascites. This is not unexpected as aging is a recognized risk for most diseases [21]. In our study, 92.1% of malignancy‐related ascites patients were > 40 years old. It has been shown that the increasing number of transforming mutations occurring in aging humans is fostered by a much more permissive environment, which allows DNA damage to occur and allows transformed cells to progress into malignancy and metastatization [21, 22]. Similarly, 95.3% of patients with liver cirrhosis in our cohort were > 40 years old. The common causes of chronic liver disease, namely MASLD, hepatitis B, or chronic alcohol consumption, result in liver damage by a pathway of chronic inflammation of the liver parenchyma, which lead to fibrosis and eventually cirrhosis [23]. This pathway of long‐term damage will take decades before the development of cirrhosis, and ascites formation will take even longer as a complication of cirrhosis‐related portal hypertension [24].

Congestive cardiac failure and chronic renal failure are also more prevalent in older adults. Approximately 1% of all persons over 40 years of age suffer from congestive cardiac failure, and it doubles with each decade of life [25, 26] due to cardiovascular risk factors which increase with age advancement [25]. The increased frequency of chronic kidney disease (CKD) among the elderly is due to the growing prevalence of established CKD risk factors including diabetes, hypertension, and cardiovascular disease (CVD) [27, 28].

Our study has also highlighted a variation in etiology of ascites between male and female genders. A higher frequency of cardiac failure and liver cirrhosis among males, compared to females, is in keeping with the epidemiology of both diseases in Malaysia [29, 30, 31]. Interestingly, this observation has been reported in Western studies too. In a study from Italy, males were more likely to suffer from liver diseases compared to females as they make up a larger proportion of cases reported for hepatitis B virus infection, alcohol‐related liver disease, non‐alcoholic fatty liver disease, cryptogenic liver disease, Wilsons disease, and hereditary hemochromatosis [32]. Males were also recorded to have a higher incidence of ascites due to congestive cardiac failure (20.3%) compared to females (12.5%). In the United States, the Framingham study reported that the incidence of heart failure was higher in males than females with an age‐standardized ratio of 1.67.

The influence of ethnicity on the etiology of ascites in the current study is a novel observation.

It appears that the most common cause of ascites among ethnic Chinese, ethnic Malays and ethnic Indians were malignancy, heart failure, and chronic liver disease, respectively (Table 2). Our study revealed that the top three malignancies which caused ascites were breast, hepatocellular, and colon carcinoma. Epidemiological studies in the Malaysian population have highlighted that all three types of cancers occur more frequently among ethnic Chinese compared with other ethnic groups [33]. A higher prevalence of Hepatitis B infection [31], particular dietary traits and a higher socioeconomic status [13] among the ethnic Chinese have been proposed to be the underlying factors for their higher rates of liver, colon, and breast cancer [34]. With regards to cardiac failure, its' causes include ischemic heart disease, arrhythmias, and uncontrolled blood pressure. Previous studies have demonstrated that ischemic heart disease is more common among ethnic Malays compared to other ethnic groups in Malaysia [35]. Furthermore, ethnic Malays, compared to other ethnic groups, have been shown to have higher rates of diabetes, hypertension, and smoking, all of which are known risk factors for ischemic heart disease [36]. Hence, it is not surprising that cardiac failure induced ascites was more prevalent among ethnic Malays, in contrast to other ethnic groups. Lastly, MASLD has been identified as the leading cause of cirrhosis in Asia [37, 38]. In Malaysia, ethnic Indians have been shown to have highest rate of both MASLD and alcoholic liver disease [31, 39]. This is likely to explain the greater proportion of liver disease‐related ascites among ethnic Indians in our study.

Despite the interesting observations regarding ascites epidemiology, this study is not without some limitations. The retrospective study design is limited by selection bias, as only patients who had undergone an ultrasound scan were included. There may have been cases with ascites who were not investigated with imaging, although this would be rare in a referral hospital like ours. Our institution is additionally a national referral center for oncology, gastroenterology, and cardiology, and the inclusion of such specialized cases may not be representative of the general population, particularly in semi‐urban or rural settings. However, the strengths of the study are the large sample size and a multi‐ethnic Asian population, making our data relevant to other populations as well. Furthermore, an objective diagnostic criteria for ascites, that is, based on imaging, enabled us to include patients who may not have had clinically detectable ascites, and hence a broader range of diseases.

In summary, we have demonstrated that malignancy and liver cirrhosis are the most common etiologies of ascites in a multi‐ethnic Asian population. An increased age (> 40 years) was associated with most diseases which caused ascites. Male patients with ascites were more likely to have liver cirrhosis and cardiac failure, compared with female adults. Ethnicity had an influence on the etiology of ascites, with a higher rate of malignancy among ethnic Chinese, greater prevalence of liver cirrhosis in ethnic Chinese and Indians, and more cardiac failure among ethnic Malays. These observations may be useful for clinicians managing adult patients with ascites in Asia, particularly for rationalizing complex investigations utilized in such patients.

## Conflicts of Interest

Dr. Sanjiv Mahadeva is an Editorial Board member of JGH Open and a co‐author of this article. To minimize bias, he has been excluded from all editorial decision‐making related to the acceptance of this article for publication.
